# Artificial Pneumo-Thorax

**Published:** 1915-01

**Authors:** A. G. Shortle

**Affiliations:** Albuquerque, New Mexico


### TEXAS MEDICAL JOURNAL

Dr. F. E. Daniel, Founder. Established July, 1885

MRS. F. E. DANIEL, - Publisher and Managing Editor

Published Monthly.—Subscription, $1.00 a Year.

VOL. XXX AUSTIN, JANUARY, 1915 NO. 7

The publisher is not responsible for views of contributors

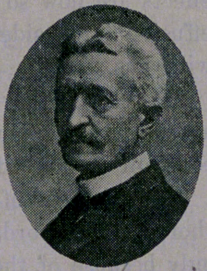

### Original Articles.

### Artificial Pneumo-Thorax.

BY A. G. SHORTLE, M. D., ALBUQUERQUE, NEW MEXICO.

The operation of artificial pneumo-thorax consists simply in the introduction of some gas, usually nitrogen or air, into the pleural cavity, allowing the lung of that side to collapse, with the result that the lung of the opposite side takes up the work for both.

The good accomplished is, of course, due to mechanical effects only. It places the diseased lung at rest, just as a splint places a tuberculous knee at rest,

This comparison is not complete, however, for it is easy to shield a diseased joint from more than occasional motion, while the lungs are expanding and contracting sixteen to twenty-two times a minute normally, not only during the day, but at night.

This rest of the organ not only permits of quicker and better scar tissue formation, but it has been shown by Shingu that there is a lymphatic stasis produced with consequent diminution in the absorption of toxins.

The collapse also allows of better drainage and the walls of cavities are so contracted that healing is much more probable.

The lessened cough and expectoration also tends to lessen the danger of extension, such as into the intestines or throat or by aspiration. Almost every practitioner has been able to see the good effects of lung collapse produced by pleurisy with effusion. Nature here does with the effusion just what we do with gas. I have seen repeatedly a tuberculous patient with high fever, cough and copious expectoration, show most marked improvement following a pleuritic effusion on the involved side. These good effects are now so universally recognized that drawing off such an effusion is considered bad practice, unless the effusion is replaced with gas.

Many of us have also seen good results follow the formation of a spontaneous pneumo-thorax; indeed, such improvement is rather constant if pus infection does not take place.

*Historical.*—Before describing the operation of induced pneumothorax a few words descriptive of its development is desirable, not only because of the natural interest one would have in its history, but also to explain why this procedure once quite popular with the phthisotherapist was for quite a while practically abandoned as of no value, and now after a lapse of fifteen years or more is more extensively used than before.

Reviewing the history briefly, then, it is known that Carson, an English physician, suggested this treatment for phthisis as far back as 1842. It was probably fortunate that he confined himself to theory, however, and did not attempt to carry out the procedure since, lacking a knowledge of asepsis, he would most certainly have infected most of his cases.

Soon after the general recognition of Lister's teaching in the matter of asepsis in surgery Forlanini, of Pavia, began to experiment on animals, collapsing their lungs first with liquids, but later changing to gases, first using oxygen, later air and finally nitrogen, selecting the last named gas because it is non-irritating and also because he thought that the 20 per cent of oxygen in the air would be absorbed more quickly than would be the case if only nitrogen was used. He began these experiments in 1882, and in 1894 first published his results in the *Muenchener Med. Wochenschrift;* this report included the results in the human as well as the animal.

In 1898, at the Denver meeting of the A. M. A., Dr. J. B. Murphy, of Chicago, reported on the results in five cases in which he had used this means. This was the first work of the kind done in America and, owing to Dr. Murphy's prominence in surgery, his endorsement of the treatment resulted in its adoption by a number of physicians in America, and for a while the method was rather extensively used.

This popularity was short lived, however, as unfavorable reports ranging all the way from slight emphysemas to air embolism began to be made. Murphy himself soon abandoned the method. This in part was occasioned by the death of Dr. Lemke, who carried out the work for him, but largely no doubt through disappointment with the results.

Forlanini must have largely abandoned the operation for a time also since he contributed nothing to the literature on the subject between 1894 and 1906.

The abandonment of this valuable procedure can be explained by a number of reasons, the chief of which are probably, first, that the operation was a dangerous one at that time, as the monometer which is so valuable in telling us when the needle is in the pleural space had not been discovered; second, it was the usual custom to collapse the lung completely the first operation, with the result that uncollapsed lung had to assume its increased burden so suddenly that it sometimes broke down under the strain if there was a lesion on the uncollapsed side; third, it was the custom with Murphy and his followers at least to collapse the lung only the one time, as a rule, with the result in most cases that the rest provided was of too short duration to be of much benefit.

To Professor Rudolph Brauer, of Marbourg, Germany (more recently of Hamburg, Germany), is largely due the credit of overcoming some of these difficulties and proving the value of the operation. His first paper was in 1904, and in 1911 he was able to report on 102 cases.

Schmidt, Sampson, Lucius, Spengler, Schriber and a number of others soon took up the work, but the introduction of the monometer by Christopher Saugman was the greatest single step, as it rendered the operation much safer and gave us an accurate method of measuring the inter-pleural pressure.

The reintroduction of this method in America is largely to be credited to Robinson and Floyd, of Boston, and to Dr. Mary Lapham, of Highlands, N. C., each of whom early in 1912 published results of cases treated by this method. M. A. Sloane, Balboni, Gekler, L. Hamman and several others, including the writer, began to use the method soon after the above.

Nitrogen was the gas almost entirely used for some years, since it is non-irritating and it was thought that the 20 per cent of oxygen in air, if it were used, would soon be absorbed. Hamman, of Baltimore, and Webb, of Colorado Springs, have shown, however, that nitrogen introduced into the pleural space soon acquires oxygen and becomes practically air, and further proved that the nitrogen sold in America, at least, almost always contained 20 per cent of oxygen, so it is largely the custom now to simply use filtered air.

*Apparatus.*—The apparatus to be recommended in my opinion is that of Robinson and Floyd, of Boston. It has several features that recommend it, but the two that are most important is, first, it is impossible to give a high pressure with this instrument; second, the large blunt needle with a beveled point makes air embolism a very unlikely event; in fact, I do not know of its occurrence when this needle was used.

The solution in the pressure bottle should contain some non-irritating antiseptic, and the air or gas should be put in fresh before each operation. We also keep an open bottle of 40 per cent formalin solution setting in the case containing the apparatus. This is to keep the exterior of the apparatus itself aseptic.

*Operation.*—All instruments should, of course, be boiled and all the usual precautions as to hands, instruments, dressings, etc., usual to aseptic surgery should be observed.

In selecting the point of incision, the first consideration is the possibility of pleuritic adhesions, which, of course, are to be avoided. This is not always simple, however, and I know from personal experience that even the X-ray is not infallible, since I have been able to get fair collapse in cases that appeared by that method to have extensive adhesions.

In the right chest I usually first try the anterior surface or the axillary space and in the left side the axillary space, unless I am convinced there are adhesions there, or that there is a larger superficial cavity, a lung abscess or other reasons for avoiding that area, but any part of the chest may be used.

Having decided upon the point for introducing the needle, the patient should be so placed as to broaden to a maximum the intercostal spaces; this can be accomplished by placing one or two pillows under the chest on the opposite side without any at their head. The skin is then washed with gasoline and then painted with tincture of iodine. Then anesthetize the skin and underlying tissue with 2 per cent novocain solution, being sure to inject the parietal pleura, for if the much-talked-of "pleural shock" ever really occurs it must be when this detail is overlooked.

With a small two-edged knife make a small incision down and through the internal intercostals in an oblique direction, the large special needle is then forced through the pleura, the oblique edge of which is held so as to strike the opposite pleura with the blunt end.

The rubber tube running to the monometer should now be attached to the needle and the stylette of the needle removed. If the needle point is rightly placed; i. e., if it has pushed through the parietal pleura and rests against the pleura covering the lung, the natural tendency of the lung to collapse exerts itself and this is recorded in the monometer by the water in the U-shaped tube being drawn up on the side connected to the rubber tube leading to the needle.

This negative pressure varies with inspiration and expiration so that the water in the monometer while showing a constant negative pressure also shows marked oscillations, moving up and down with each respiration.

It is well to emphasize here the fact that on the correct interpretation of the monometer reading depends the safety of the operation.

*Never turn on gas until you have both a negative pressure and oscillation* is a rule that if observed will make this operation one practically devoid of danger.

You may have oscillation without a negative pressure, or you may have a negative pressure without oscillation, but be sure you have *both* before turning on the gas.

Failing to get one or both of these, the point of the needle should be slowly turned and moved from side to side to free it from any obstruction and the stylette reintroduced to force out a possible piece of tissue or blood clot, the tube leading to the monometer examined for obstruction and still failing to get the negative pressure and oscillations recorded we must conclude that we have our needle point in a pleural adhesion so a new point must be selected in a different part of the chest, in hopes of finding a place free of adhesion.

Three or four failures at different parts of the chest will justify one in thinking the case inoperative. We find we are unable to introduce gas in about 20 per cent of the cases.

Getting both a negative pressure and oscillations, h wever, we now carefully turn on the gas, allowing 50 to 100 c.c. to flow in, then shut it off and again try the monometer; if a strong negative pressure is still recorded 200 to 300 c.c. more gas may be introduced, but if we find the negative pressure greatly reduced, or even a positive pressure recorded, we interpret this as meaning that we have simply entered a pocket fenced off by adhesions, so it is well to try at another point in hopes of finding a free pleura.

Supposing a free pleura space is found, 300 to 600 c.c. of gas may be introduced, the needle withdrawn and a small piece of aseptic gauze or cotton strapped over the skin puncture.

The first operation proving successful, the succeeding ones are quite simple, since the pleural space is now established. One can now use simply a fairly coarse aspirating needle, as there is now little danger of injuring the lung.

*Selection of Cases.*—It must be remembered, in deciding on the advisability of using lung collapse in a given case, that the greatest good to be accomplished is through rest of the collapsed lung and of necessity this means more work for the uncollapsed lung, so we must first decide whether or not we think the better lung can be trusted to do not only its own work, but that of its more extensively diseased fellow.

Naturally, the ideal case is the one with the involvement limited to one lung, but it is seldom we find a really unilateral case among advanced consumptives.

As we will show later, however, many of our most happy results have been achieved in marked bilateral cases. It is not easy to explain just why giving rest to a badly involved lung improves it and making the one less involved work harder improves that one also.

This may be in part at least explained by the wonderful improvement in the patient's general condition through the lessened absorption of toxins with consequent lowered fever and other symptomatic improvement consequent to the collapse of the worst lung; this probably more than neutralizes the bad effects of the increased work on the uncollapsed lung. Kuhn has shown that under certain conditions either rest or exercise to the lung may prove beneficial, and in artificial pneumo-thorax we are using both treatments in the same case. Whatever the theoretical explanation may be, however, we know by experience that the working lung often improves very rapidly. So far we have only used this treatment in unfavorable cases, and not until other means of treatment had failed in our estimation.

Brauer and others have warned us to avoid cases with other tuberculous complications, such as laryngitis, enteritis, nephritis, etc., but in my own experience I have found the effects on these complications were beneficial more often than the reverse.

From my own experience I should give the following rules for selection of cases:

Any case having one good lung, or one that is comparatively so, and

(a) Having the opposite lung extensively involved, particularly if there is cavity formation, or

(b) Having the opposite lung only moderately involved but getting progressively worse and not responding to the usual modes of treatment, or

(c) Having a moderate involvement the patient on account of finances could not afford the usual long lay off from work necessary to cure (Balboni, Chitty and others have reported good results among patients treated while earning a livelihood), or

(e) Having severe or recurrent hemoptysis.

Doubtful cases would be those coming under one of the above classifications, but where the collapsed lung was so extensively involved as to cause doubt as to inability to stand up under the work, and in all cases that would be otherwise suitable but rendered doubtful as complications such as tuberculous enteritis, laryngitis, nephritis, etc., or organic heart trouble.

Under the heading of "doubtful" might also be placed spontaneous pnuemo-thorax and bronchi-ecstasis.

Forlanini has given the following rules for the selection of cases:

1. Uncomplicated cases of slow or subacute course, and with pleura free from adhesions, regardless of the degree of lesion.

2. The same with such adhesions as may be removed by artificial pneumo-thorax.

3. Bilateral phthisis not running an acute course and with lesions on both sides not far advanced.

(To be continued.)

